# CDK4/6 Inhibitors and Arthralgia: A Single Institution Experience

**DOI:** 10.3390/medsci9020042

**Published:** 2021-06-05

**Authors:** Angeliki Andrikopoulou, Oraianthi Fiste, Kleoniki Apostolidou, Efthymia Skafida, Christos Markellos, Michalis Liontos, Anastasios Kyriazoglou, Meletios-Athanasios Dimopoulos, Flora Zagouri

**Affiliations:** Department of Clinical Therapeutics, Alexandra Hospital, School of Medicine, 11528 Athens, Greece; aggandrikop@med.uoa.gr (A.A.); ofiste@med.uoa.gr (O.F.); apostolidoukl@gmail.com (K.A.); efiskafida@med.uoa.gr (E.S.); chrismarkellos@med.uoa.gr (C.M.); mlionto@med.uoa.gr (M.L.); tassoskyr@gmail.com (A.K.); mdimop@med.uoa.gr (M.-A.D.)

**Keywords:** CDK4/6 inhibitors, arthralgia, musculoskeletal, toxicity, breast cancer

## Abstract

Background: Aromatase inhibitors (AIs) are associated with musculoskeletal pain in one third (20–47%) of breast cancer patients. Recently, CDK4/6 inhibitors have emerged as a new therapeutic approach in hormone receptor (HR)-positive breast cancer. While hematological and gastrointestinal toxicities are frequently reported during treatment with CDK4/6 inhibitors, musculoskeletal symptoms are less commonly encountered. Methods: Herein, we present a retrospective study of 47 breast cancer patients who received CDK4/6 inhibitors along with endocrine therapy in our department between 01/01/2018 and 01/09/2020. Results: Median age at diagnosis was 58 years (29–81). Median duration of treatment was 8.76 months (SD: 7.68; 0.47–30.13 months). Median PFS was 24.33 months (95% CI; 1.71–46.96). Overall, toxicity was reported in 61.7% of the cases (29/47). Arthralgia was reported in 6.4% (3/47) of the patients. Hematological toxicity was reported in 51.1% (24/47) of the patients. Neutropenia was the main hematological toxicity observed (86.8%; 22/47) along with anemia (4.3%; 2/47), thrombocytopenia (2.1%; 1/47), and leukopenia (4.2%; 1/24). Conclusions: Though our data reflect a small sample size, we report a reduced arthralgia rate (6.4%) during treatment with CDK4/6 inhibitors compared with that reported in studies of AIs (20–47%).

## 1. Introduction

Aromatase inhibitor-induced musculoskeletal syndrome (AIMSS) is a syndrome commonly encountered by breast cancer patients treated with aromatase inhibitors (AIs). Even though third generation AIs have been widely implemented in clinical practice, musculoskeletal symptoms often lead to treatment discontinuation. Approximately one third of breast cancer patients receiving AIs (20–47%) report AI-induced joint symptoms, most commonly arthralgia and myalgia. The prevalence of musculoskeletal symptoms varies within different studies according to the type of aromatase inhibitor administered, previous chemotherapy treatment, prior treatment with tamoxifen, and body mass index [1,2]. Eventually, up to 20% (2–24%) of patients receiving AIs withdraw from treatment because of musculoskeletal pain [3,4]. Median onset of symptoms is anticipated at two months of treatment, with a peak occurrence at six months [5,6]. The underlying mechanism of AI-related arthralgia is not well-defined, although it has been related to estrogen deprivation [1,2]. As treatment with AIs is widely applied in hormone-receptor (HR)-positive breast cancer, the incidence of arthralgia caused remains of major importance.

Today, cyclin-dependent kinase 4/6 (CDK4/6) inhibitors have emerged as new treatment options in the management of advanced or metastatic breast cancer. In February 2015, the Food and Drug Administration (FDA) granted accelerated approval to CDK4/6 inhibitor Palbociclib for the treatment of metastatic HR-positive, human epidermal growth factor receptor 2 (HER2)-negative breast cancer based on results of the PALOMA-1 Phase II study [7]. Ribociclib was then approved by FDA in March 2017 in combination with letrozole for the treatment of postmenopausal women with HR-positive, HER2-negative advanced breast cancer based on the MONALEESA-2 trial [8]. The third CDK4/6 inhibitor that received FDA approval in metastatic breast cancer was abemaciclib and is currently under investigation as an adjuvant treatment in patients with high-risk node-positive early breast cancer in MonarchE trial [9]. CDK4/6 inhibitors restore the repressive effect of retinoblastoma (RB)-associated protein on the E2F family of transcription factors attenuating cell cycle progression [10]. Cyclin-dependent kinases 4/6 (CDK4/6) catalyze the hyperphosphorylation of RB protein and disrupt its onco-suppressor effect. CDK4/6 inhibitors prevent the inactivation of RB by cyclin-dependent kinases 4/6 and reinstate its suppressive effect on cell passage through the G1-S checkpoint. Phase III trials have demonstrated that CDK4/6 inhibitors offer prolonged progression-free (PFS) and overall survival (OS) in patients with metastatic HR-positive, HER2-negative breast cancer compared with endocrine therapy [11]. A recent meta-analysis confirmed that the combination of CDK4/6 inhibitors and endocrine treatment achieves a significantly improved PFS (hazard ratio (HR) 0.54, 95% confidence interval (CI) 0.50–0.59, *p* < 0.00001) and OS (HR 0.77, 95% CI 0.69–0.85, *p* < 0.00001) compared with endocrine therapy alone in HR-positive, HER2-negative advanced breast cancer [12]. Collectively, these findings led to the implementation of CDK4/6 inhibitors in current clinical practice.

The toxicity profile of CDK4/6 inhibitors is well-defined based on Phase III trials. Hematologic toxicities are the most common adverse events for palbociclib or ribociclib. Approximately 85% and 76% of breast cancer patients treated with palbociclib or ribociclib, respectively, experience neutropenia of any grade [13]. Grade 3 or 4 neutropenia is reported in up to 60% of patients receiving palbociclib or ribociclib. As for abemaciclib, diarrhea is the toxicity most commonly reported (85%), although typically low-grade [13].

Interestingly, arthralgia of any grade was observed in 18%, 29%, and 14% of patients treated with palbociclib, ribociclib, or abemaciclib, respectively [13]. In addition, Phase III studies revealed a lower arthralgia rate in the arm of the CDK4/6 inhibitor plus endocrine therapy compared with the control arm [14,15,16]. Considering this deviation from the expected incidence of arthralgia, we evaluated arthralgia incidence in patients treated with CDK4/6 inhibitors in our institution. Here, we present our experience regarding joint symptoms in breast cancer patients treated with CDK4/6 inhibitors.

## 2. Materials and Methods

This is a single-institute, retrospective study, which was carried out in the Oncology Unit of Clinical Therapeutics Department of University of Athens in Alexandra General Hospital. The study was performed in accordance with the 1964 Helsinki Declaration and was approved by the institutional ethics committee on November 30, 2020 (Ethic Code: 56941). Medical records of patients who were treated with CDK4/6 inhibitors in the adjuvant or metastatic setting between 01/01/2018 and 01/09/2020 were retrospectively reviewed. All subjects gave written informed consent. Data were collected through a single institution database that consists of clinicopathological, treatment-related, and survival data. Breast cancer patients that received CDK4/6 inhibitors in the adjuvant setting were also included.

### Statistics

Descriptive statistics were used to assess the clinicopathological parameters of the patients. Disease progression was defined as the time between the initiation of treatment with CDK4/6 inhibitors and the date of local or distant disease recurrence. Progression free survival (PFS) was calculated from the initiation of treatment with CDK 4/6 inhibitors until disease progression (PD) or last follow-up in metastatic breast cancer patients. The distribution of PFS was estimated using the Kaplan–Meier method. Statistical analysis was performed with SPSS 24.0 statistical software.

## 3. Results

### 3.1. Patients Characteristics

Between January 2018 and September 2020, 47 women received treatment with CDK4/6 inhibitors in our department. Median age at diagnosis was 58 years (29–81). There were 21.3% (10/47) premenopausal, 66% (31/47) postmenopausal, and 12.8% (6/47) perimenopausal women. Overall, 53.2% (25/47) of women were diagnosed with early breast cancer, while 46.8% (22/47) of women were presented with de novo metastatic disease. The tumor was estrogen receptor (ER)-positive in 95.7% (45/47) of the cases and progesterone receptor (PR)-positive in 91.5% (43/47) of the cases. Breast cancer was hormone-receptor positive (ER- or/and PR-positive) in all cases (100%) and human epidermal growth factor receptor 2 (HER2)-negative in all cases. Most breast tumors were grade II (51.1%; 24/47), while 38.3% (18/47) were grade III. Adjuvant chemotherapy was administered in 92% (23/25) of women with early-stage breast cancer, while 80% (20/25) of women with early disease received adjuvant hormone therapy. In this population, 76% (19/25) of women had progressive disease and received CDK4/6 inhibitors, while the rest of them received CDK4/6 inhibitors in the adjuvant setting. Overall, CDK4/6 inhibitors were administered as adjuvant treatment in 12.8% (6/47) of the cases, as 1st line in 53.2% (25/47) of the cases and as 2nd line treatment in 29.8% (14/47) of the cases.

### 3.2. Survival Analysis

**Figure 1:**
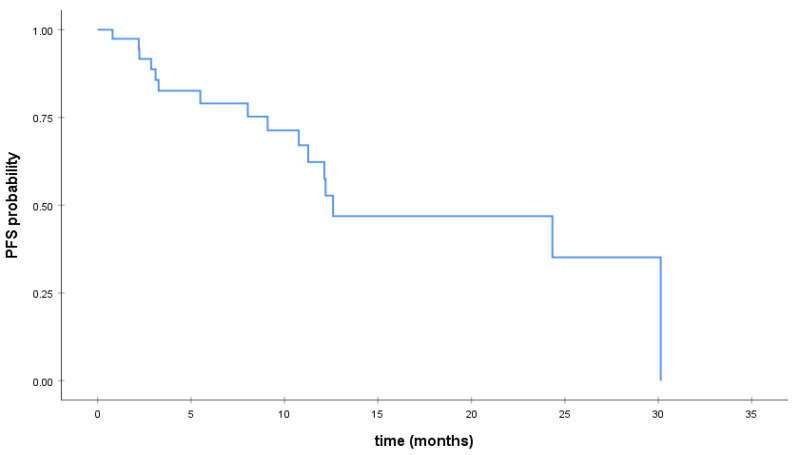
Kaplan–Meier curve for progression-free survival (PFS) in Stage IV breast cancer patients.

Overall, 12.8% (6/47) of women received abemaciclib, 63.8% (30/47) received palbociclib, and 23.4% (11/47) of women received ribociclib. CDK4/6 inhibitors were administered in combination with fulvestrant (34%), letrozole (59.6%), or tamoxifen (6.4%). Overall, 34% (16/47) experienced progressive disease while on treatment with CDK4/6 inhibitors. In addition, 38.3% (18/47) of patients discontinued treatment with CDK4/6 inhibitors. Reasons of discontinuation were disease progression (88.9%), hematological toxicity in two cases (11.1%), and hyperkalemia in one case (5.6%). Median duration of treatment was 8.76 months (SD: 7.68; 0.47–30.13 months). Overall, median PFS was 12.6 months (95% CI; 1.34–23.85) for patients treated with CDK4/6 inhibitors for metastatic disease. The Kaplan–Meier curve for PFS is depicted in Figure 1.

### 3.3. Association of CDK4/6 Inhibitors with Arthralgia

Overall, toxicity was reported in 61.7% of the cases (29/47) (Table 1). Arthralgia was reported in 6.4% (3/47) of the patients, including two women with arthralgia and one woman reporting arthralgia and muscle pain in the lower extremities. None of the patients presenting with arthralgia discontinued treatment with CDK4/6 inhibitors. Of note, hematological toxicity (neutropenia, anemia) was reported in 51.1% (24/47) of the patients. Dose reduction was reported in 25.5% (12/47) of women treated with CDK4/6 inhibitors, while treatment was interrupted in 51.1% (24/47) of women.

## 4. Discussion

We evaluated treatment-associated musculoskeletal symptoms in patients treated with CDK4/6 inhibitors in our department. The incidence of arthralgia reported was 6.4%, which is lower than the incidence reported in Phase III trials. Two of the patients that experienced arthralgia received CDK4/6 inhibitors in combination with aromatase inhibitors and one with fulvestrant. Interestingly, none of the patients experiencing joint pain discontinued treatment.

Arthralgia has been more frequently reported in patients treated with palbociclib compared with the other two approved CDK4/6 inhibitors [13]. A meta-analysis of PALOMA trials reported an incidence of 24.5% of any grade arthralgia in palbociclib-treated patients, however only 0.8% of grade 3/4 arthralgia [14]. According to the results of the MONALEESA trials, ribociclib is associated with a 24–28% rate of any grade arthralgia, which is reduced compared with the hormonotherapy only arm in postmenopausal women [15,16]. Consistently, more severe arthralgia (grade 3/4) was observed as less than 1% in treatment with ribociclib. As previously mentioned, abemaciclib is expected to induce joint pain less frequently. The MONARCHE-2 trial reported an incidence of 11.6% of any grade arthralgia in abemaciclib plus fulvestrant treatment group and 0.2% of grade 3/4 arthralgia, while 12.54% of patients suffered from any grade arthralgia in the MONARCHE-3 trial [17,18,19]. In the majority of studies, the musculoskeletal symptom rate is reduced in the CDK4/6i-treated population. Table 2 summarizes arthralgia incidence in Phase III trials evaluating CDK4/6 inhibitors. The reason for this difference is not yet defined.

In our study, arthralgia incidence was 6.4% in patients treated with CDK4/6 inhibitors either in the adjuvant or in the metastatic setting. This incidence is lower than the one described in Phase III trials [14,15,16]. Firstly, there is an interstudy heterogeneity between Phase III studies. Consistently with our findings, the MONARCHE PLUS trial (NCT02763566) reported an arthralgia rate of 5.85% among patients receiving CDK4/6 inhibitors in combination with AIs and 6.73% among those receiving CDK4/6 inhibitors plus fulvestrant [20]. In addition, the number of patients enrolled in our study was limited. Moreover, real world data may differ from the toxicities reported in Phase III studies (Table A1). Some adverse events are often underreported when not inducing a major discomfort in the patient. Discrepancies between AE reporting in clinical trials often emerge from the underreporting of low-grade AEs [21]. Underreporting in the medical record or administration of concomitant medication that could affect the arthralgia incidence remains a possible bias in our study. Finally, there is no distinction between the different types of CDK4/6 inhibitors in our study. We report the arthralgia induced by CDK4/6 inhibitors in general; however, there are differences between the toxicity induced by each one. For instance, we included patients receiving abemaciclib, which is related to lower joint toxicity. However, we still report a reduced incidence of treatment-related joint pain in treatment with CDK4/6 inhibitors.

Multiple mechanisms of AI-induced arthralgia have been reported. The main mechanism proposed is via estrogen deprivation [2]. Aromatase inhibitors may decrease estrogen levels by over 85% in postmenopausal women [22]. Estrogen receptors (ERα and ERβ) are expressed in human articular chondrocytes and modulate the chondrocyte turnover in the bone microenvironment [23]. This finding supports the bone-protective role of estrogens in the joint. Consistently, menopause leads to major estrogen decrease and musculoskeletal pain similar to that reported with AIs. In addition, exogenous estrogen or SERM administration suppresses the progression of cartilage erosion exerting a chondroprotective effect on articular cartilage. Indeed, hormone replacement therapy results in a reduction up to three-fold of osteoarthritis incidence [24,25]. Estrogen deprivation leads to the increased production of RANKL by stromal cells and the reduction of osteoprotegerin, thus leading to increased osteoclastogenesis and bone breakdown [26]. Moreover, aromatase inhibitors may cause autoimmune-related joint pain presenting with autoimmune antibodies (ANA) and rheumatoid factor (RF), while cases of rheumatoid arthritis have been reported [27,28,29]. Indeed, estrogen deficiency stimulates T-cell secreted inflammatory cytokines interleukin-6 (IL-6) and tumor necrosis factor-a (TNF-a), which regulate osteoclast activity [30]. Eventually, approximately half of patients develop AI-induced arthralgia and 20% discontinue treatment early because of intolerance [1,6,31]. The onset of symptoms can occur at any time point, although most symptoms develop within 1–3 months of treatment initiation [6,30,31].

CDK4/6 inhibitors regulate the passage of cells in the S phase by maintaining the repressive effect of RB protein on the E2F family of transcription factors [10]. During the G1 phase, Rb protein forms an inactive complex with E2F transcription factors which prevents the expression of key genes required for cell cycle progression. In the hyperphosphorulated state, Rb protein loses its onco-suppressor function and releases E2F transcription factors to drive the expression of E2F-responsive genes. Apart from their role in cell cycle progression, E2Fs possess multiple functions in angiogenesis, tumor metastasis, and inflammation [32,33]. It has been shown that E2F2 upregulates IL-1 and TNF-a in rheumatoid arthritis (RA) synovial fibroblasts [34]. E2F2 binds to the promoters of STAT1 and activates the PI3K/AKT/NF-κB pathway regulating the expression of IL-1 and TNF-a, which lead to joint damage and RA development [34,35]. Aberrant expression of inflammatory markers like IL-1 and TNF-a mediates the destruction of joint cartilage. Thus, CDK4/6 inhibitors could attenuate the E2F2-mediated joint inflammation by retaining the Rb-induced suppression of E2Fs. Moreover, CDK6 functionally interacts with the NF-kB subunit and is recruited to the promoters of many NF-kB target inflammatory genes [36,37]. Knockdown of either CDK4 or CDK6 suppressed multiple IL-1-induced genes like Il-8 and Il-6. It was shown that inflammatory gene expression induced by IL-1 or TNF-a occurs in a CDK6-dependent pathway [36,37]. Similarly, CDK4/6 inhibitors may inhibit the expression of numerous cytokine-mediated inflammatory genes disrupting the joint inflammation process.

Regardless of the mechanism, CDK4/6 inhibitors have been shown to exert an anti-inflammatory mechanism in the joint microenvironment. CDK4/6 inhibitor palbociclib inhibited the proliferative phase of rheumatoid arthritis in an animal model, especially when combined with TNF-a or IL-6 blockers [38]. Palbociclib exerted an antiarthritic effect by inhibiting synovial hyperplasia and interfering with the formation of the pannus [38]. Another study also demonstrated the antiarthritic effect of CDK4/6 inhibitor palbociclib [39]. Treatment with palbociclib suppressed fibroblast-like synoviocyte proliferation in the joint synovium by interfering with the p16-RB pathway. Other CDK4/6 inhibitors have also demonstrated an antiarthritic effect by suppressing matrix metalloproteinase-3 production by synovial fibroblasts and osteoclastogenesis of macrophages [40].

Collectively, there is evidence that CDK4/6 inhibitors possess a protective role in treatment-related arthritis. Here, we here report a reduced rate of treatment-related arthralgia (6.4%) in patients receiving CDK4/6 inhibitors compared with hormone monotherapy treatment (20–47%) [1,2,5].

## Figures and Tables

**Table 1:** Adverse events of treatment with CDK4/6 inhibitors.

Overall Toxicity N (%)	*Yes*	*29 (61.7)*
	No	15 (31.9)
	Not reported	3 (6.4)
*Hematological toxicity N (%)*	Yes	24 (51.1)
	No	20 (42.6)
	Not reported	3 (6.4)
*Type of hematological toxicity N (%)*	Neutropenia	22 (86.8)
	Anemia	2 (4.3)
	Neutropenia/thrombocytopenia	1 (2.1)
	Neutropenia/leukopenia	1 (2.1)
*Arthralgia N (%)*	Yes	3 (6.4)
	No	42 (89.4)
	Not reported	2 (4.3)

**Table 2:** Baseline characteristics of patients.

Patient Characteristics	All Patients (47)	
Age, median, range	58	(29–81)
Menopausal status, N (%)	Perimenopausal	6 (12.8)
	Premenopausal	10 (21.3)
	Postmenopausal	31 (66)
Stage at initial diagnosis, N (%)	Non-metastatic	25 (53.2)
	Metastatic	22 (46.8)
Histology, N (%)	IDC	39 (83)
	ILC	5 (10.6)
	Mucinous	1 (2.1)
	IDC/ILC	1 (2.1)
	Not reported	1 (2.1)
ER status, N (%)	Positive	45 (95.7)
	Negative	2 (4.3)
PR status, N (%)	Positive	43 (91.5)
	Negative	4 (8.5)
Grade, N (%)	I	2 (4.3)
	II	24 (51.1)
	III	18 (38.3)
	Not reported	3 (6.4)
Adjuvant hormonotherapy, N (%)	Yes	20 (80)
	No	4 (16)
	Not reported	1 (4)
Adjuvant chemotherapy, N (%)	Yes	23 (92)
	No	1 (4)
	Not reported	1 (4)
Adjuvant radiation therapy, N (%)	Yes	18 (72)
	No	6 (24)
	Not reported	1 (4)
Disease progression, N (%)		20 (42.6)
CDK4/6 inhibitor, N (%)	Abemaciclib	6 (12.7)
	Palbociclib	30 (63.8)
	Ribociclib	11 (23.4)
Concomitant treatment, N (%)	Fulvestrant	16 (34)
	Letrozole	28 (59.6)
	Tamoxifen	3 (6.4)
Treatment duration, median, range	8.76	0.47–30.13
Dose reduction, N (%)	Yes	12 (25.5)
	No	33 (70.2)
	Not reported	2 (4.3)
Dose interruption, N (%)	Yes	24 (51.1)
	No	20 (42.6)
	Not reported	3 (6.4)
Treatment discontinuation, N (%)	Yes	18 (38.3)
	No	27 (57.4)
	Not reported	2 (4.3)
Reason of discontinuation, N (%)	Hematological toxicity	2 (4.3)
	Hyperkalemia	1 (2.1)
	PD	16 (34)
Disease progression on CDK4/6 inhibitor, N (%)		16 (34)
Progression-free survival (PFS), median, range	24.3	1.71–46.96

## Data Availability

Data presented in our study can be found in the patients’ archives that are safely stored in our Institution. The datasets generated during the current study are available from the corresponding author upon request.

## Appendix A

**Table A1:** Clinical trials of CDK4/6 inhibitors and the treatment-related arthralgia rates in breast cancer.

Clinical Trial	Trial Number	Treatment Arms	Sample Size (Size per Arm)	Median Age	Median Follow-Up	PFS	OS	Arthralgia (%)
PALOMA-1	NCT00721409	palbociclib plus letrozole vs. letrozole plus placebo	165 (84/81)	63/64	64.7	20.2/10.2 (HR:0.488; *p* = 0.0004)	37.5/34.5 (HR: 0.897; *p* = 0.281)	27.7/18.2
PALOMA-2	NCT01740427	palbociclib plus letrozole vs. letrozole plus placebo	666 (444/222)	62/61	23	24.8/14.5 (H: 0.58; *p* < 0.001)	42.1/34.7 (*p* = 0.06)	33.3/33.8
PALOMA-3	NCT01942135	palbociclib plus fulvestrant vs. fulvestrant plus placebo	517 (345/172)	57/56	44.8	9.2/3.8 (HR: 0.422; *p* < 0.0001)	34.9/28 (HR: 0.81; *p* = 0.09)	13/16.3
MONALEESA-2	NCT01958021	ribociclib plus letrozole vs. placebo plus letrozole	668 (334/334)	62/63	26.4	25.3/16 (HR: 0.568)	42.5/28.7; H: 0.746	27.5/28.8
MONALEESA-3	NCT02422615	ribociclib plus fulvestrant vs. placebo plus fulvestrant	726 (484/242)	63.4/62.8	39.4	20.5/12.8 (HR: 0.593; *p* < 0.001)	57.8%/45.9% at 42 months	23.8/26.6
MONALEESA-7	NCT02278120	ribociclib plus tamoxifen/NSAI plus goserelin vs. placebo plus tamoxifen/NSAI plus goserelin	672 (335/337)	42.6/43.7	19.2	23.8 vs. 13 (H: 0·55, *p* < 0·0001)	NR	29.85/27.3
MONARCHE-2	NCT02107703	abemaciclib plus fulvestrant vs. placebo plus fulvestrant	669 (446/223)	59.3/61.1	19.5	16.4/9.3 (HR: 0.553; *p* < 0.001)	NR	11.6/14.3
MONARCHE-3	CT02246621	abemaciclib plus anastrozole or letrozole vs. placebo plus anastrozole or letrozole	493 (328/165)	63/63	26.8	28.2 vs. 14.7; (H: 0.54; *p* < 0.001)	NR	17.43/20.5
MONARCHE PLUS	NCT02763566	abemaciclib plus NSAI vs. placebo plus NSAI or abemaciclib plus fulvestrant vs. placebo plus fulvestrant	463 (207/99/104/53)	56/59/55/58)	26	Not reached vs. 14.7; H: 0.49	56/30 (*p* < 0.0001)	5.8/13.1 6.7/5.6
